# A detailed characteristics of bias associated with long runs of homozygosity identification based on medium density SNP microarrays

**DOI:** 10.7150/jgen.39147

**Published:** 2020-04-07

**Authors:** Tomasz Szmatoła, Artur Gurgul, Igor Jasielczuk, Weiwei Fu, Katarzyna Ropka-Molik

**Affiliations:** 1 University Centre of Veterinary Medicine, University of Agriculture in Kraków, Al. Mickiewicza 24/28, 30-059 Kraków, Poland.; 2 National Research Institute of Animal Production, Department of Animal Molecular Biology, Krakowska 1, 32-083 Balice, Poland; 3 College of Animal Science and Technology, Northwest A&F University, Yangling, Shaanxi 712100, China

**Keywords:** runs of homozygosity, autozygosity, microarray, next generation sequencing

## Abstract

In the present study, runs of homozygosity (ROH) detected with the use of a standard bovine 54k single nucleotide polymorphism (SNP) genotyping assay and two different ROH detection approaches, based on 50 (M1) or 15 (M2) consecutive SNPs, were compared with results of whole genome sequencing. Both microarray-based methods accurately recognised medium-sized ROH, however, it was found that M2 method seemed to better than M1 identify short ROH, but highly overestimated their number, leading to numerous false positive calls. Moreover, long ROH identified with microarray data tended to break into shorter segments in sequencing data because of the presence of regions with high heterozygosity within the ROH sequences. This may indicate, that these long ROH are formed by closely positioned shorter homozygous segments that may be of older origin or may be created by two similar but not identical haplotypes, showing minor internal recombination signs. Such finding also suggests that at least some of the results of previous studies in regard to long ROH may be biased leading to inaccurate estimations of genomes autozygosity via ROH classification into length categories.

## Introduction

In recent years, the availability and continues reduction of costs of new high-throughput analytical methods such as genome sequencing and high-density SNP arrays has led to obtaining precise information about specific chromosomal segments which includes regions of extended homozygosity. These long homozygous stretches of the genome, so called runs of homozygosity (ROH), may be formed due to mating of related individuals which led to transition of haplotypes that are identical by descent 1,2,3,4. In human genetics, information obtained from ROH is inter alia used to identify the susceptibility of individuals to recessive diseases 5,6. However, in animal genetics it is more commonly used as a measure of genomic inbreeding (FROH) or for genome-wide autozygosity mapping 2,7,8,9.

In the latest years, most ROH analysis were maintained on medium or high-density SNP arrays - in the case of bovine - either 54k or 777 k chip. Despite a large number of studies, many of the parameters required to evidence ROH differ in the literature. In general, a minimal ROH length of 1 Mb and a 1 Mb maximum gap between SNPs is assumed in most studies performed with a 54 k chip. However, a number of consecutive homozygous SNPs required within ROH vary and ranges from 15 to 50. The number of heterozygotes allowed within ROH also differs between studies, ranging from 0 to 1 for the 54k chip, to even 16 for long ROH (above 16Mb) in the case of the 777k chip 8,10. These differences and large distances allowed between SNPs hampers unambiguous and comparable ROH detection as well as stimulate to investigate what percentage of ROH detected with genotyping technologies is true and properly reflecting autozygosity events. To contribute to this knowledge, in this study we attempt to compare ROH detected with standard 54 k arrays with results of whole genome sequencing - based on two different ROH detection thresholds.

## Materials and Methods

The material of the study was genomic DNA obtained from semen of a Polish Red bull. DNA was either genotyped with use of Illumina BovineSNP50 BeadChip (standard Infinium Ultra protocol) or sequenced-by-synthesis using Illumina HiScanSQ system. Across the genome, SNPs were detected using Freebayes software 11 and filtered to retain only those of the highest quality possible - with genotype quality higher than 20 and minimal adjusted coverage of 10. A detailed description of sequencing and SNP detection method is presented in Supplementary File 1. In the case of microarray data, two computational approaches were used: one that requires a minimum of 50 consecutive homozygous SNPs to assign ROH and focus mainly on long homozygous segments (M1) and another that requires 15 consecutive SNPs (M2) and allows detection of a higher number of short ROH. In both approaches, we did not allow any heterozygotes within ROH up to 16 Mb of length and allowed for one heterozygote in ROH above 16 Mb (based on 8 and calculated by assuming a genotype error of 0.2% as suggested by Howrigan et al. 12). Regarding missing values, we allowed 0 for 1-2 and 2-4 Mb category, one in 4-8 Mb, two in 8-16 Mb and four in >16Mb category. In the case of sequencing data, similar approaches were used, but corrected (multiplied) by the number of SNPs used for analysis across the genome - S1 requiring a minimum of 5150 consecutive homozygous SNP (and allowing 27 heterozygotes) and another, S2, that required 1545 SNPs (while allowing 8 heterozygotes). The minimal number of consecutive homozygous SNPs used in this approaches was comparable between arrays and sequencing and was derived from a simple proportion (103 times more SNPs in sequencing data after exclusion of X, MT chromosomes and unmapped contigs, resulting in 5,456,266 SNPs vs 52,886 SNPs from microarray), maintaining minimal requirement regarding 1Mb ROH length. In addition, the number of heterozygotes allowed within ROH in these approaches was derived from the mutual genotyping error observed between SNP chip and sequencing data calculated for a fraction of 20,612 common SNPs. This genotyping error was low and equalled to 0.54%. A cgaTOH software 13 was used for all ROH identification steps. The detected ROH were compared between separate approaches by evaluation of their number, lengths, genomic positions and size of overlapping segments.

The obtained results concerning gaps breaking the longest microarray-based ROH into shorter segments in sequencing data were further validated in two other animals of Holstein breed which were sequenced in the course of another study 14 and deposited in the Bovine Genome Variation Database and Selective Signatures (http://animal.nwsuaf.edu.cn/code/index.php/BosVar/loadByGet?address[]=BosVar/Download/varData.php). The .vcf files containing information about the variation in their genomes were processed in the same way as for Polish Red bull and included in total 4 250 029 SNPs for the first and 4 438 040 SNPs for the second animal. Microarray-based ROH (M1 approach) were detected based on reduced sequencing dataset involving markers common for both array and sequencing methods with cgaTOH software settings established individually per animal as described above.

## Results and Discussion

**Figure 1:**
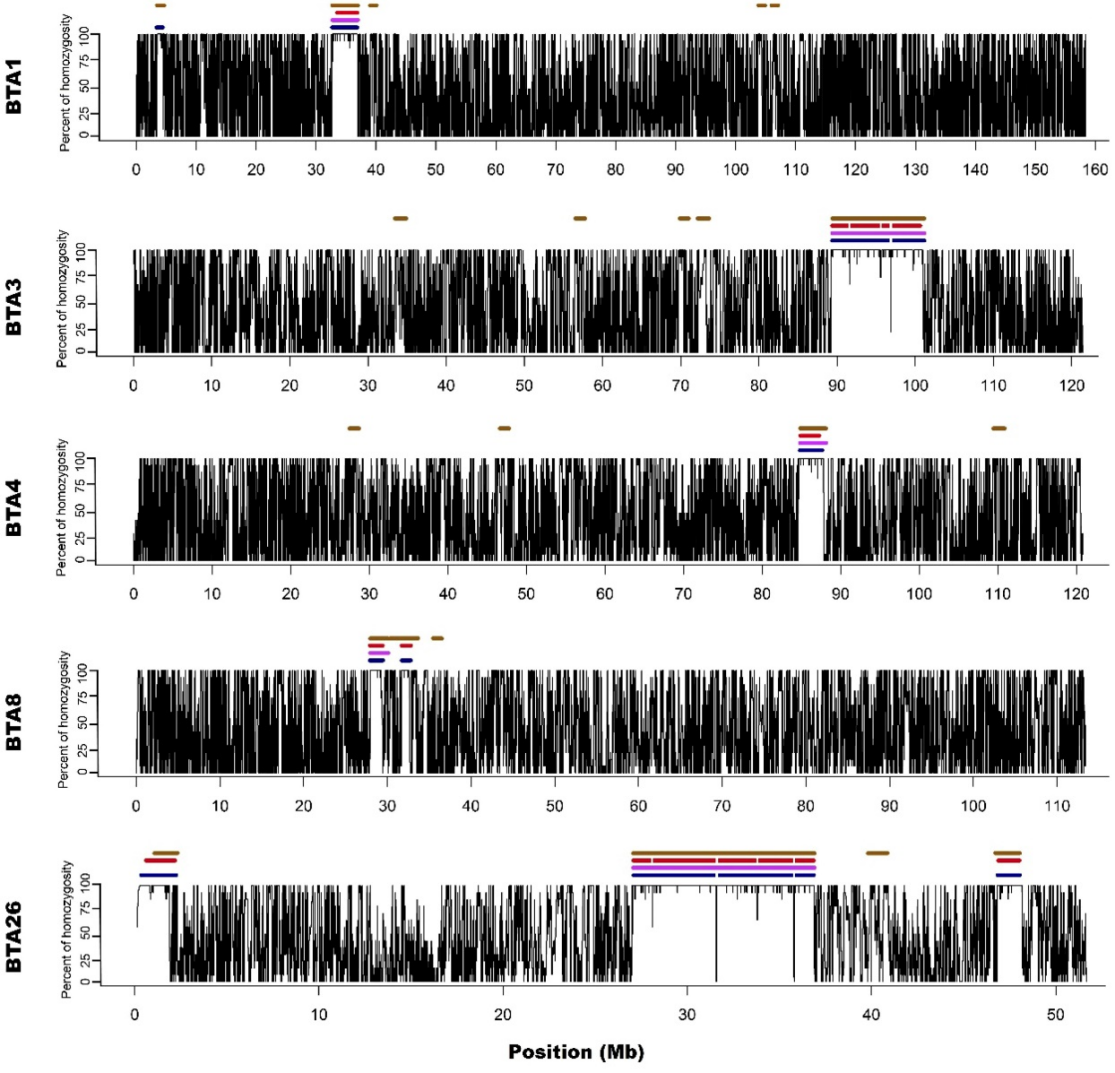
Visualization of ROH identified with all used methods. *Black lines for each chromosome represent average homozygosity in a sliding window of 15 consecutive SNPs. Blue colour represents S1 approach; violet represents M1 approach; red represents S2 approach and brown represents M2 approach.*

**Figure 2:**
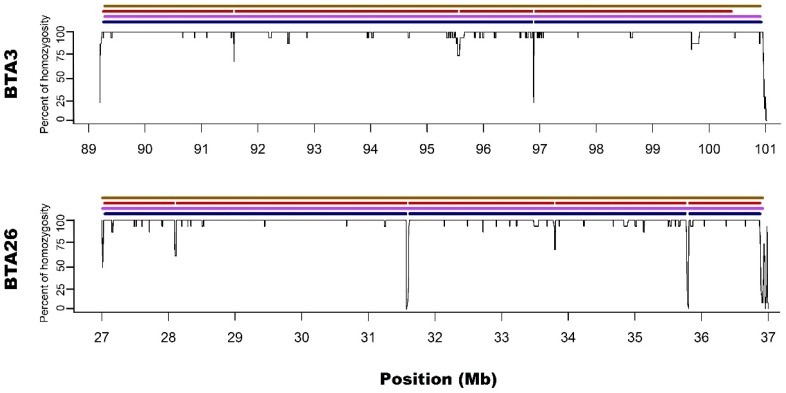
Zoom on the long ROH identified on chromosome 3 and 26 for all used methods. *Black lines for each chromosome represent average homozygosity in a sliding window of 15 consecutive SNPs. Blue colour represents S1 approach; violet represents M1 approach; red represents S2 approach and brown represents M2 approach.*

For all approaches and data types (array/sequencing), ROH appeared on 5 different autosomes (1,3,4,8 and 26). The number of ROH and the sum of ROH lengths was the highest in the case of M2 approach and the lowest for the M1 method. Both sequencing-based approaches showed intermediate values. The detailed ROH positions along with their lengths are presented in Supplementary File 2, graphically in Figure 1 and 2, while the ROH statistics are presented in Supplementary File 3.

M1 approach (using 50 consecutive SNPs) enabled to identify 5 ROH - three above 4 Mb of length and two spanned 3.3 and 2.1 Mb, while corresponding S1 method showed 13 ROH with size from 1 to 4.5 Mb. Three of the long ROH detected with the M1 method were broken into 2-3 shorter segments in the case of S1. Regarding M1 vs. S1, it was noted that microarray-based ROH overlapped with 75.6% of all ROH segments identified by sequencing-based approach, while in the case of sequencing method, it overlapped with 94.7% all ROH identified with the use of microarray data. The observed difference is associated with the presence of short ROH identified based on sequencing data that were not detected in microarray-based approach.

Microarray-based M2 method (focusing on shorter ROH segments by requiring only 15 consecutive SNPs) has led to detecting 21 ROH - three long ones that were above 4 Mb and 18 with various short lengths ranging from 1 to 3.3 Mb. Corresponding sequencing-based approach (S2) showed 17 ROH that were from 1 to 4 Mb in length. Those three long ROH detected with M2 were identical with those identified by the M1 method and in a similar way, they were broken into 2-5 segments in S2 approach. In the case of M2 vs. S2, we found that microarray-based ROH overlapped with 88.1% of all ROH identified by sequencing-based approach, while in the case of sequencing data, it overlapped with 64.4% of all ROH identified with the use of microarrays. It is clear that M2 overlapped a higher percentage of ROH detected by sequencing approach than M1 due to the identification of a higher number of short ROH. However, it also resulted in detection of a high number of false short ROH (which were not detected using any sequencing-based approach). This false-positive short ROH caused that only 64.4% of all ROH segments overlapped between S2 and M2 approaches.

Regarding all overlaps between all applied approaches (presented in Supplementary File 4) it is clear that M2 approach in general overlapped better with sequencing approaches than M1. However, when it was tested how M2 method is described by three other methods, the percentage of overlaps was the lowest. This proves the statement that M2 appears to overestimate the number of short ROH leading to the identification of many false-positives.

In regard to the gaps breaking the longest microarray-based ROH into shorter segments in sequencing data, it was noted that S1 method detected 3 gaps: one on BTA3 and two on BTA26; while S2 had 7 gaps: three on BTA3 and 4 on BTA26. Moreover, all of the gaps identified by S1 were also present in S2. The ROH gaps had various lengths from 894 bp to 23306 bp and were formed by a different number of heterozygous SNPs in close vicinity (from 4 to 21). The gaps identified by S1 approach are especially interesting since they were formed by 12, 15 and 21 heterozygous SNPs, positioned in a close vicinity - all of them having high read coverage (from 12 to 21 with a mean of 16.5) and genotype quality (mean=237.6; SD=148.2), suggesting that they are unlikely genotyping errors. Also, calculated GC content within analysed regions (Table 1) and a sequence characteristics did not suggest any read mapping issues associated with repetitive or low complexity sequences. Regarding the results of S2 method, it identified all of the gaps that were observed in S1 with an addition of 4 smaller gaps only visible in this approach. These additional smaller ROH gaps were formed by a lower number of heterozygous SNPs and may be either true biological observations or artificial gaps that arose due to the used method of ROH identification. This suggests that S2 method, similarly like M2, may not be that suitable for ROH detection with sequencing data. The details about gaps in ROH along with their statistics are presented in Table 1.

The presence of highly heterozygous regions within long ROH detected with microarrays was additionally confirmed based on whole genome sequencing of other two animals of Holstein breed obtained in the course of another study and by an independent sequencing laboratory. Within the longest ROH detected in these animals with M1 approach, several highly heterozygous gaps were detected (Supplementary File 5). This observation confirms that the presence of short highly heterozygous gaps within long ROH detected with microarrays is a common phenomenon which is likely independent on technical genotyping aspects or animal-specific features. Moreover, in the research of 15 similar observations were noted for populations characterized be recent inbreeding such as Isle Royale and Mexican wolves: long ROH segments were interspersed with regions of high heterozygosity.

In previous research, various authors suggested that 50k chips may not be dense enough to detect short ROH 1,7,16. According to Purfield et al. 1, only 27.7% of all ROH from the category 1-5 Mb were detected in comparison to BovineHD BeadChip. However, in general longer ROHs were detected with a similar sensitivity as in the case of HD chip. Ceballos et al. 16 compared the results of ROH analysis for different SNP arrays and whole genomes sequencing of low coverage. The Authors indicated that short and medium length ROH may be only correctly identified by microarrays of above 300k SNP size. Moreover, the boundaries of ROH obtained from SNP microarray data compared with WGS data will be fuzzier and not that precise.

In addition, Ferenčaković et al. 7 presented that 54k SNP assays overestimated the number of ROH that were shorter than 4 Mb. When comparing the results obtained in this study, it is clearly visible that microarray-based approach requiring only 15 SNPs in ROH (M2) tended to identify some short ROH that are false positives and are not present in sequencing data. Longer ROH, however, tend to overlap between 54k assay and sequencing data regardless of the approach used. What is interesting, both approaches based on microarray data show long ROH, above 9 Mb on chromosome 26 and 11 Mb on chromosome 3. With sequencing data, for both approaches, these long ROH break into shorter segments (visually presented in Figure 1 and Figure 2). This may indicate, that these long ROH are formed by closely positioned shorter homozygous segments that may be of older origin. Moreover, it suggests that at least some of the long ROH presumably being accurately identified with SNP arrays in different studies are in fact formed by several separate chromosomal segments. Such dense genomic co-localization of these segments is, however, difficult to explain, but putatively may be connected with selection pressure on certain gene variants, resulting in presence of long and common haplotypes at the specific locus. Nevertheless, in our previous research, identifying ROH patterns in various breeds of cattle maintained in Poland 4, we did not find any ROH islands in Polish Red cattle corresponding directly to the long ROH or their possible breakpoints found in this study. The other cause of this observation may be a presence in the animal genome of two long and similar, but recombinant haplotypes with an older than assumed coancestry.

In various research, it has been established that in animal genomics, ROH may be used as a tool to estimate inbreeding by calculating the portion of the genomes covered in ROH (FROH) 6,15. Strong to moderate correlations between FROH and FPED (calculated using pedigrees) for various cattle breeds with a different pedigree depth 7,9 were observed. The differences may arise because of a number of factors, including pedigree depth and accuracy, population sample size, lengths of ROH used for calculations or even used correlation test. The possible nature of long ROH detected in this study may also bias ROH-based inbreeding coefficients (by the classification of the detected long ROH to different length group) and could partially explain the lower concordance between FROH and data obtained with the use of animal pedigree observed in some individuals 9. The breaks in the long ROH bias FROH values calculated for ROH above certain thresholds which leads to the decline of FROH values. This is visible in our data, presented in Supplementary File 6, where for example for S2 method FROH values drop to 0 for ROH above 4 Mb. We also hypothesize that at least some of the long ROH detected with the use of microarrays, may be in fact composed of several shorter ROH formed by similar, but recombinant haplotypes. However, we base this statement on a small number of long ROH and there is a need for further research on a higher number of samples to prove this observation. Moreover, in this study, we confirmed that using 15 consecutive SNPs for ROH detection with 54k chip assay may result in overestimation of a number of short ROH.

## Supplementary Material

Supplementary information, figure, and tables.

## Figures and Tables

**Table 1:** The detailed characteristics of gaps found within long ROH.

**S1 method**								
Gap in adjacent ROH regions			Gap length (bp)	Neighbouring heterozygous SNPs in the gap	GC content in the gap (%)	GC content in the whole chromosome	Mean adjusted gap coverage	Adjusted gap coverage SD
Chromosome	Start (kb)	End (kb)						
3	96894	96897	3278	12	35.44	41.80	18.60	5.59
26	31581	31603	23306	21	44.21	42.77	16.25	3.87
26	35808	35811	3144	15	37.66	42.77	14.66	2.31
**S2 method**								
Gap in adjacent ROH regions			Gap length (bp)	Neighbouring heterozygous SNPs in the gap	GC content in the gap (%)	GC content in the whole chromosome	Mean adjusted gap coverage	Adjusted gap coverage SD
Chromosome	Start (kb)	End (kb)						
3	91581	91582	1009	4	48.12	41.80	14.80	2.30
3	95575	95583	7573	4	57.53	41.80	14.29	2.21
3	96894	96897	3278	12	35.44	41.80	18.60	5.59
26	28115	28116	894	6	42.79	42.77	15.50	3.83
26	31580	31603	23306	21	44.21	42.77	16.25	3.87
26	33806	33808	2630	5	47.08	42.77	18.17	3.19
26	35808	35811	3144	15	37.66	42.77	14.66	2.31
