# Supplementary material for: A detailed characteristics of bias associated with long runs of homozygosity identification based on medium density SNP microarrays
Source: J Genomics. 2020 Apr 7;8:43–8. doi: 10.7150/jgen.39147 (PMC7171384; doi:10.7150/jgen.39147)
Supplement: Supplementary file 1 — Supplementary information, figure, and tables. [file jgenv08p0043s1.pdf]

Supplement File 1

Preparation of libraries and sequencing protocol

Sherlock AX kit (A&A Biotechnology) was used to purify DNA from semen of a single Polish Red bull. Then, the preparation of libraries was maintained with the use of Nextra DNA Sample Prep kit followed by Ampure XP (Beckman Coulter) size selection step (0.7x protocol) which led to obtaining libraries with DNA insert sizes ranging from 160 to 560 bp. TruSeq Paired-End Cluster Generation Kit v3 (Illumina) was used to generate clusters on the Illumina v3 flowcell using cBot station. The paired-end (2x 100 bp) sequencing protocol was performed on the HiScanSQ system with the TruSeq SBS Cycle Sequencing Kit v3 (Illumina).

Sequencing data analysis

The obtained reads (100 bp length) were checked for quality with the use of FastQC [17] software and then filtered with Flexbar software [18] to remove reads of low quality (--pre-trim-phred 20 and --min-read-length 70). The alignment against Bos Taurus genome (UMD 3.1) was performed with BWA software [19] with default settings which led to approximately 86% of reads being mapped to the reference. Then, the obtained bam file (16.5x genome coverage) was sorted and the duplicates were removed with samtools [20]. Variants were called with Freebayes software [11] with default settings which led to detection of 7,369,096 SNPs with the mean coverage of 17. After filtration (genotype quality higher than 20 and minimal adjusted coverage of 10) 5,286,424 SNPs were left for analysis.

SNP genotyping

Illumina's Bovine50SNPv2 assay was used to assess genotypes of approximately 54 k SNPs according to Infinium Ultra protocol. SNPs with no chromosomal positions were removed from further analysis.

Supplementary File 2. Detailed ROH positions along with their lengths. M1- microarray based approach of ROH identification one; M2- microarray based approach of ROH identification two; S1- sequencing based approach of ROH identification one; S2- sequencing based approach of ROH identification two.

**Chr**	**S1**			**M1**			**S2**			**M2**		
	Start (kb)	Stop (kb)	Length (kb)	Start (kb)	Stop (kb)	Length (kb)	Start (kb)	Stop (kb)	Length (kb)	Start (kb)	Stop (kb)	Length (kb)
1	3 464	4545	1081	32738	37017	4278	33583	35660	2077	3541	4755	1213
1	32 700	35411	2711				35664	36907	1242	32738	37017	4278
1	35416	36910	1493							39053	40110	1057
1										103728	104842	1114
1										105908	107009	1101
3	89207	96894	7686	89233	100982	11748	89207	91581	2374	33403	44847	1444
3	96897	100998	4100				91582	95575	3992	56473	57705	1232
3							95583	96894	1311	69753	70931	1178
3							96897	100463	3566	72067	73526	1458
3										89233	100932	11748
4	84632	87485	2852	84649	87984	3335	84706	86081	1375	27168	28384	1216
4							86083	87102	1018	46334	47539	1204
4										84649	87984	3335
8	27919	29473	1553	27975	30129	2153	27932	29448	1515	27975	30129	2153
8	31701	32816	1115				31701	32804	1102	30320	31409	1088
8										31518	33668	2150
8										35434	36489	1055
26	4	1932	1928	26994	36959	9965	276	1856	1580	726	2001	1274
26	27031	31580	4549				27025	28115	1089	26994	36959	9965
26	31603	35808	4205				28116	31580	3463	39880	40959	1079
26	35811	36922	1111				31603	33806	2202	46878	48223	1345
26	47000	48219	1219				33808	35808	2000			
26							35811	36917	1106			
26							47037	48201	1164			

Supplementary File 3. ROH statistics for all used computational methods. M1- microarray based approach of ROH identification one; M2- microarray based approach of ROH identification two; S1- sequencing based approach of ROH identification one; S2- sequencing based approach of ROH identification two.

ROH Statistic	ROH detection Method			
	**S1**	**M1**	**S2**	**M2**
Number of ROH	13	5	17	21
Number of ROH above 4 Mb	4	3	0	3
Mean of ROH lengths (kb)	2738.8	6295.8	1877.4	2461.3
Minimum length of ROH (kb)	1081	2153	1018	1055
Maximum length of ROH (kb)	7686	11748	3992	11748

Supplementary File 4. The percent of mutual overlaps between ROH found using different detection methods.

	S1	M1	S2	M2	Mean
S1	-	94.7%	98.8%	69.7%	87.73%
M1	75.6%	-	76.9%	54.9%	69.13%
S2	96.4%	85.4%	-	64.4%	82.07%
M2	91.9%	100%	88.1%	-	93.33%
Mean	87.97%	90.05%	87.85%	63.00%	-

Supplementary File 6. Validation of the occurrence of highly heterozygous gaps within long ROH detected with the use of microarrays in two other animals of Holstein breed.

Black line represents average homozygosity in a sliding window of 20 consecutive SNPs. Dark blue color represents ROH detected with M1 approach; red arrows point to the highly heterozygous regions of unknown origin within long ROH. These regions in most cases are not detected (disregarded) by microarray-based approach suggesting continuity of the ROH segment.

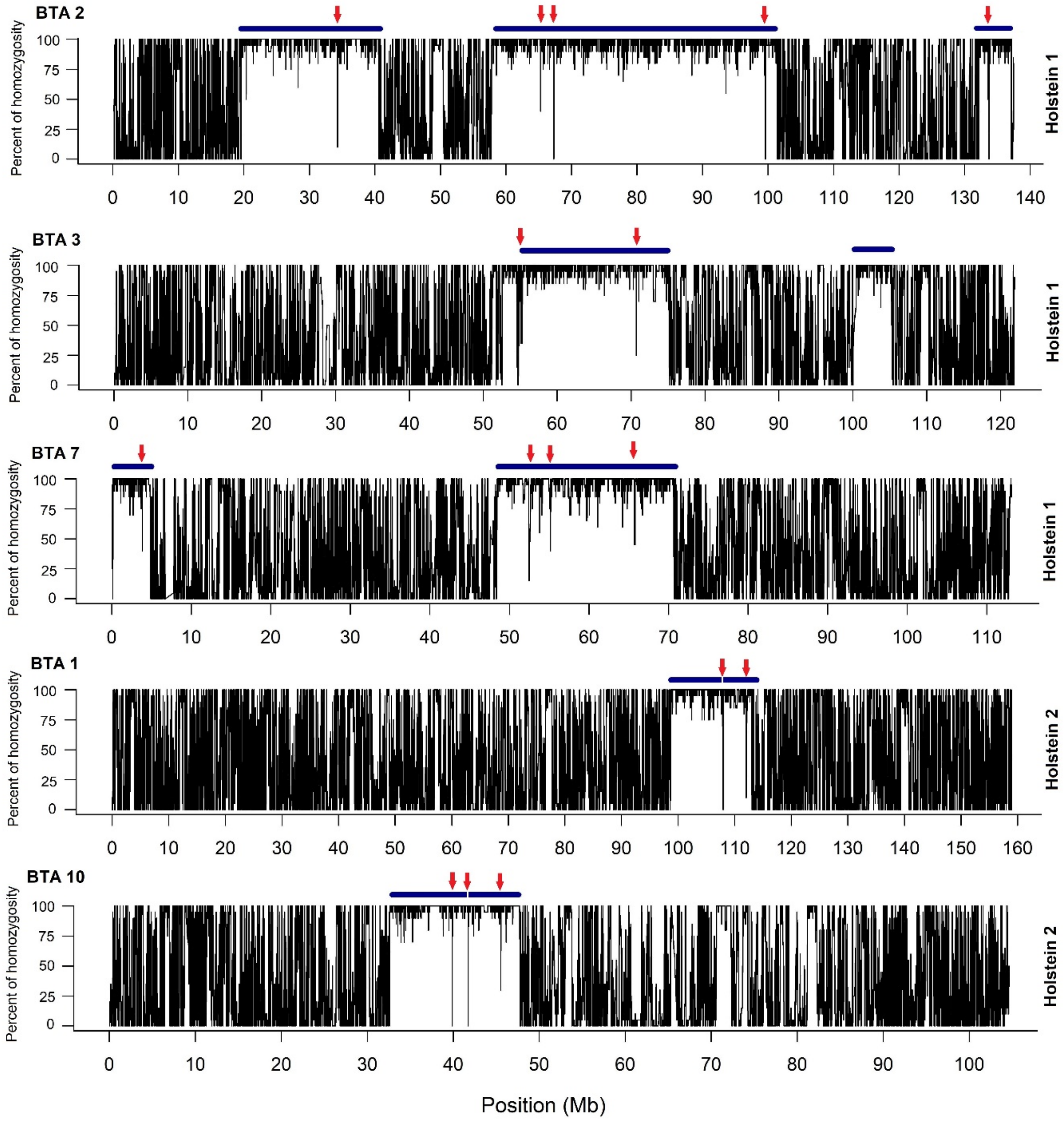

Supplementary File 5. F_{ROH} for all used methods in selected ROH length categories.

Method	F_{ROH} ROH above thresholds (Mb)			
	**1+**	**2+**	**4+**	**8+**
S1	0.014242	0.010442	0.008216	0.008216
M1	0.012592	0.012592	0.010396	0.010396
S2	0.012766	0.00703	0	0
M2	0.020675	0.013452	0.010396	0.010396

Conflicts of Interest: None of the authors have any relationships, financial or otherwise, with people or organizations that could have inappropriately influenced this work. The authors declare that they have no competing interests.
